# A Rare Hybrid Skin Cyst of the Scalp With Pilar and Apocrine Features

**DOI:** 10.7759/cureus.62071

**Published:** 2024-06-10

**Authors:** Brian Shih, Mariamma Joseph, Qi Zhang, Madison T Gray

**Affiliations:** 1 Medicine, Western University, London, CAN; 2 Pathology and Laboratory Medicine, London Health Sciences Centre, London, CAN

**Keywords:** apocrine cyst, dermoid cyst, hybrid cyst, scalp mass, dermatopathology, neuropathology

## Abstract

Benign epithelial skin cysts containing multiple components of the folliculo-sebaceous apocrine unit are only rarely reported in the literature. Here, we describe a 12-year-old girl who presented with a cystic mass on the vertex of her scalp. Upon resection, it showed a hybrid benign skin cyst with interesting histological features of both pilar and apocrine differentiation. The clinicopathological and imaging findings of this unusual skin cyst, successfully managed by a plastic surgeon and neurosurgeon, are described. Pathologists and clinicians should be aware of this type of skin cyst rarely encountered in their clinical practice.

## Introduction

Benign epithelial cutaneous cysts of ectodermal origin are commonly encountered in clinical practice and are often clinically indistinguishable. Cyst location and imaging characteristics may help to characterize these lesions; however, histologic evaluation is the gold standard for final diagnosis. Dermoid cysts are congenital malformations and consequently are most frequently identified during infancy or adolescence, whereas epidermal and pilar cysts are often acquired and can be seen in any age group [1]. The benign nature of these lesions can warrant conservative management; however, surgical excision is often performed for a number of reasons such as cosmetic appearance, risk of intracranial epidural extension (for scalp location), and risk of rupture leading to infection. In this report, we present a 12-year-old girl who underwent surgical resection for a cyst on the scalp with both pilar and apocrine features.

## Case presentation

A 12-year-old girl with a history of isolated unilateral renal hypoplasia presented with a soft, painless, non-mobile midline scalp lesion. The lesion was 3 cm in diameter, showed progressive growth, and was tender only with headgear. There was no history of trauma. The initial partial excision showed only adipose tissue and was identified as a nevus lipomatosus superficialis.

During an attempted complete excision by a pediatric surgeon, a grossly fatty lesion and an underlying fluctuant bulge were identified. Due to the concern of meningocele with incomplete closure of the anterior fontanelle, the mass was not immediately excised.

**Figure 1:**
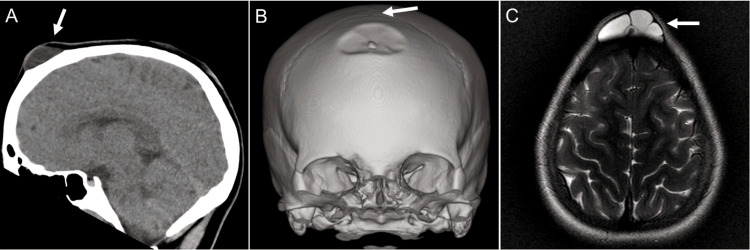
Radiographic examination of the lesion (A) A sagittal CT scan of the head demonstrates a soft tissue lesion (arrow) overlying the frontal bone; (B) Bone window reconstructions demonstrate indentation of the frontal bone which remains intact. The lesion is distant from bregma (arrow), the site of the now-closed anterior fontanelle; (C) T2-weighted MRI demonstrates the lobulated, T2-hyperintense lesion (arrow), distant from the brain

A subsequent computed tomography (CT) scan revealed complete closure of the anterior fontanelle (Figure 1A); however, there was a persistent 3 x 3 x 1.5 cm soft tissue mass with indentation of the frontal bone inferior to bregma (Figure 1B). The mass was located deep within the midline frontal scalp with underlying frontal bone remodeling and potentially a small osseous channel. No osseous defect, erosion, or demineralization was observed. The favored radiographic differential diagnoses were dermoid or epidermal cysts. Magnetic resonance imaging (MRI) confirmed the differential, demonstrating a lobulated, extracranial mass within the frontal scalp which appeared hyperintense on T2-weighted images (Figure 1C). Imaging characteristics were inconsistent with fatty material and did not support the presence of an epidermoid cyst.

The patient’s brain was structurally normal, and no intracranial communication was found with this lesion. Due to ongoing concerns, the lesion was surgically explored by a neurosurgeon, and the cyst was resected without complications. When the cyst was opened intraoperatively, no solid contents were seen, and clear fluid was noted.

**Figure 2:**
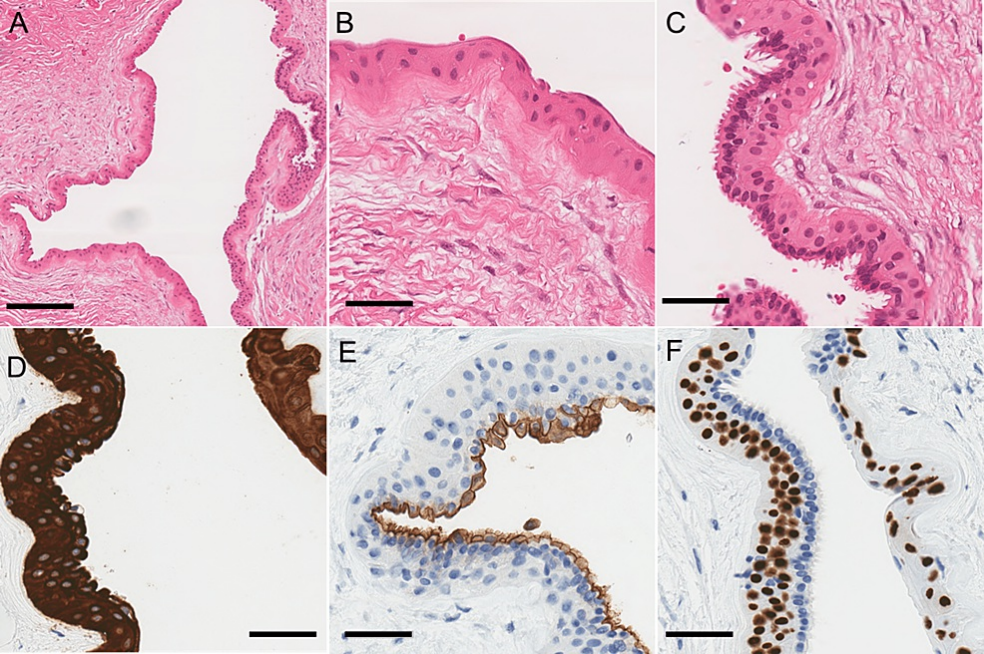
Histologic examination of the lesion (A)-(C) H&E shows the dual populations of cyst-lining cells, both pilar (B) and apocrine (C); (D) Both cell populations stain for high-molecular-weight cytokeratin; (E) CEA specifically labels the apocrine population; (F) Basal cells are labeled by p40 Scale bars = 200 µm (A) and 50 µm (B)-(F) CEA: carcinoembryonic antigen

The gross examination showed a partly collapsed deep cutaneous cyst (2.5 x 2.0 x 0.7 cm) with no connection to the epidermis. Histology revealed a cystic lesion lined by benign multilayered epithelial cells and supported by a thick fibrous capsule with dense collagen (Figure 2A). The cyst lining consisted of two populations of cells. The cyst was mostly lined by swollen stratified squamous epithelium without a granular layer consistent with a pilar cyst (Figure 2B). The second population of cells showed apocrine glandular features that had an inner layer of elongated columnar cells with apocrine snouts indicating “decapitation secretion” on histology (Figure 2C). Underlying swollen stratified squamous epithelium of the pilar cyst remained. Skin adnexal structures, such as hair follicles, sebaceous glands, and sweat glands, were not seen on the cyst wall. A diagnosis of a hybrid cutaneous cyst with pilar and apocrine features was made. All cyst lining cells were diffusely positive for CK19, high-molecular-weight cytokeratin, and CK AE1/AE3 (Figure 2D). The apocrine epithelial population was immunopositive for CK7, carcinoembryonic antigen (CEA), and epithelial membrane antigen (EMA), with the diastase-treated periodic acid Schiff (PAS) stain highlighting a brush-border appearance at the luminal surface (Figure 2E). Basal cells were immunoreactive for p40 and p63 (Figure 2F). All cyst lining cells were negative for the progesterone receptor, smooth muscle actin, muscle-specific actin, gross cystic disease fluid protein-15 (GCDFP-15), and mammaglobin. Clinical follow-up at four months was unremarkable, with complete healing of the incision and no recurrence of the cyst.

## Discussion

The term hybrid cyst was initially coined to define cutaneous cysts that originate from a hair follicle and contain characteristics of both infundibulum (epidermal cyst) and isthmus (pilar/trichilemmal cyst) portions of a hair follicle [1]. This term has since been expanded to include any type of cyst that has a combination of two or more components of the folliculo-sebaceous-apocrine unit, which may include infundibulum, isthmus, or lower portion (hair bulb) of the hair follicle, sebaceous gland, and apocrine gland [2]. Although most reported hybrid cysts are those that contain a combination of epidermal and pilar elements, there have been rare reports of cysts such as hybrid pilomatricoma and epidermal cysts, as well as hybrid eruptive vellus hair cyst and steatocystoma, all derived from a hair follicle [2,3]. However, hybrid pilar and apocrine cysts are only rarely encountered in the literature.

There are many approaches for categorizing benign cutaneous cysts. One proposed is by the cystic origin in the folliculo-sebaceous-apocrine unit [4]. Through identifying unique clinical characteristics, histology, and cytokeratin expression profiles, cysts can also be classified based on their histogenesis.

**Figure 3:**
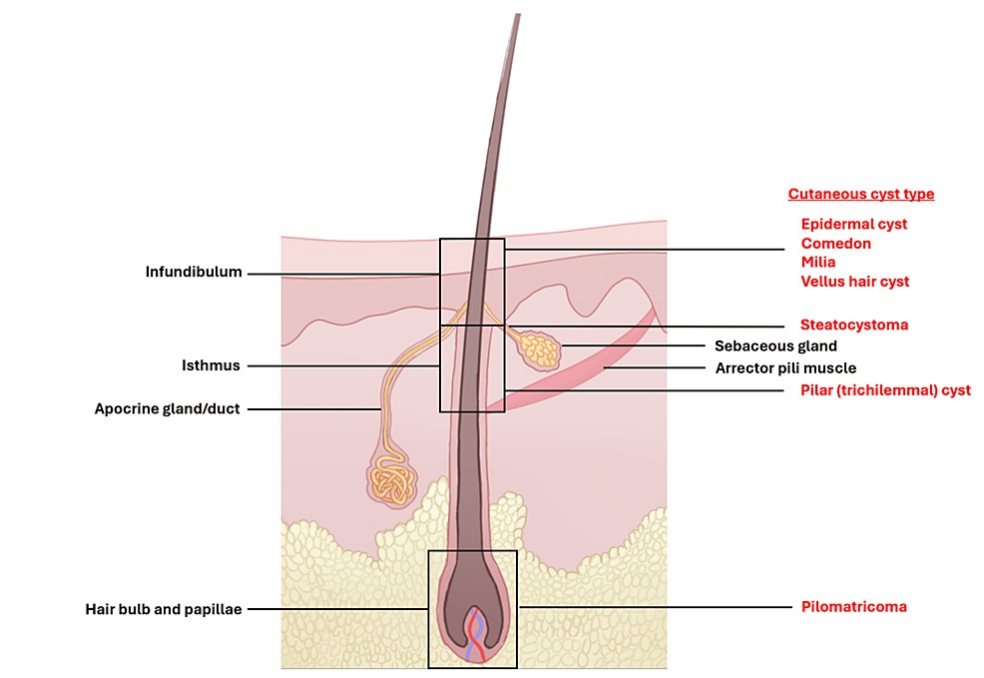
Diagram of the follicular structural unit with cutaneous cysts organized by their origin in the folliculo-sebaceous-apocrine unit.

Epidermal (epidermoid) cysts are a commonly encountered cysts that can occur anywhere on the body, originate from the infundibulum of the hair follicle, and are lined by stratified squamous epithelium with a preserved granular layer [2,4]. Pilar (trichilemmal) cysts occur most frequently on the scalp, are derived from the isthmus (external root sheath) of the hair follicle, and are lined by swollen stratified squamous epithelium with abrupt keratinization lacking a granular layer [2,4]. Both these cysts contain laminated, eosinophilic keratin. Apocrine cysts are much rarer benign cysts that arise from apocrine sweat glands [5]. Histologically, these cysts consist of a luminal layer of columnar cells showing apocrine snouts and a peripheral layer of flattened myoepithelial cells. Steatocystoma multiplex originates from sebaceous ducts, and pilomatricoma arises from the hair bulb [4]. A diagram demonstrating the organized follicular structural unit including the folliculo-sebaceous-apocrine components and arrector pili muscle is presented (Figure 3).

Hybrid cysts, exhibiting characteristics from multiple components of the folliculo-sebaceous-apocrine structural unit, are relatively rare. These have been best described on eyelids [6]. Another case on the scalp, in a 42-year-old male, displayed both trichilemmal type epithelium and a transition to columnar apocrine type epithelium [7]. Another instance of a hybrid cyst, featuring epidermal and apocrine attributes, was described in the axillary region of a 45-year-old woman, demonstrated primarily infundibular type hair follicle epithelium, with some areas displaying apocrine epithelium, and a distinct transition between the two types of epithelium [8]. These documented cases provide valuable insights into the rare occurrence and diverse manifestations of hybrid cutaneous cysts.

The scalp is known for its abundance of deeply situated terminal hair follicles, making it a common site for various types of cutaneous cysts [9]. While apocrine glands are not typically found in the scalp, the theory proposing a common embryological origin for the folliculo-sebaceous-apocrine unit [10] offers a plausible explanation for the hybrid nature of cysts observed in this region. According to this theory, the shared developmental origin of these skin appendages can result in the occurrence of hybrid cysts displaying a combination of features from different components of the folliculo-sebaceous-apocrine unit. Therefore, the presence of diverse adnexal combinations in hybrid cysts found on the scalp aligns with this embryological theory.

A broad differential is required for benign cystic lesions on the scalp in the pediatric population, including cysts of congenital, infectious, neoplastic, and traumatic origin. Meningoceles is a congenital neural tube anomaly that arises within herniations of intracranial tissue through skull defects [11,12]. These occur most commonly in the occipital region; however, they can also arise in the basal and frontoethmoidal regions of the skull. Dermoid and epidermal cysts are common causes of pediatric skull tumors [13]. Dermoid cysts often occur along cranial sutures or the anterior fontanelle, histologically containing stratified squamous epithelium and adnexal structures on the cyst wall. Although both cysts are lined by squamous epithelium and contain keratin, epidermal cysts distinctly lack adnexal structures [13,14].

MRI plays a key role in demonstrating and differentiating deep cystic lesions of the scalp in pediatric populations. Meningoceles appear as a pouch of meningeal membranes filled with cerebrospinal fluid [12]. Epidermal cysts have characteristic restricted diffusion on diffusion-weighted imaging and are isointense to CSF on T1- and T2-weighted images [15]. In comparison, dermoid cysts do not have restricted diffusion and are typically hyperintense on T1-weighted images with variable signals on T2-weighted images [15].

Our patient presented with a lobulated, extracranial cystic mass on the scalp, which could be an epidermal cyst, pilar cyst, or dermoid cyst on imaging. There was no restricted diffusion nor keratin-like contents in the cyst lumen. The midline frontal location of the mass is not commonly associated with meningoceles, and the clear cyst fluid noted during excision was potentially retained sweat and not cerebrospinal fluid. Histologically, the mass was identified as a benign hybrid cyst, with swollen stratified squamous epithelium characteristic of a pilar cyst and a population of elongated columnar cells with apical snouts indicative of apocrine features. In the absence of adnexal structures on the cyst wall, the histologic features are not supportive of a dermoid cyst.

Management of deep cystic lesions on the scalp depends on the cyst type and origin. Meningoceles are often diagnosed prenatally using methods such as ultrasound and maternal alpha-fetoprotein (AFP), while MRI is the preferred modality for pre-operative imaging and diagnostic confirmation [12]. Neurosurgical intervention usually provides a definitive cure as delaying treatment increases the risk of meningocele rupture and infection. Epidermal, pilar, and dermoid cysts can be managed conservatively; however, these cysts may enlarge and become symptomatic by compressing surrounding structures in addition to raising cosmetic concerns [12]. Furthermore, cutaneous cysts carry the risk of rupture and infection, with dermoid cysts potentially progressing to malignancy in rare circumstances, if left untreated. Excisions of dermoid, epidermoid, and pilar cysts are considered relatively safe, with a study of 234 patients undergoing cyst excision finding no major complications nor recurrence after three months [12]. However, recommended management often involves a neurosurgical intervention to avoid potential complications in pediatric populations. In this case, complete excision was performed by a pediatric neurosurgeon due to the concern of the general surgeon of incomplete closure of fontanelle and meningocele.

## Conclusions

In summary, we report a case of the hybrid cutaneous cyst, with pilar and apocrine differentiation, on the scalp of a pediatric patient. This case demonstrates the importance of a thorough physical examination and possibly neuroimaging in pediatric patients to uncover any underlying calvarial defects and carry out a definite treatment protocol. Careful pathologic examination of cutaneous cysts can reveal unusual and interesting hybrid histologic features as seen in this case.
